# Supplementary material for: Structural basis for ligand promiscuity and high signaling activity of Kaposi’s Sarcoma-associated Herpesvirus-encoded GPCR
Source: Nat Commun. 2025 Sep 25;16:8403. doi: 10.1038/s41467-025-63457-4 (PMC12462484; doi:10.1038/s41467-025-63457-4)
Supplement: Supplementary file 1 — Supplementary Information [file 41467_2025_63457_MOESM1_ESM.pdf]

Figure S1

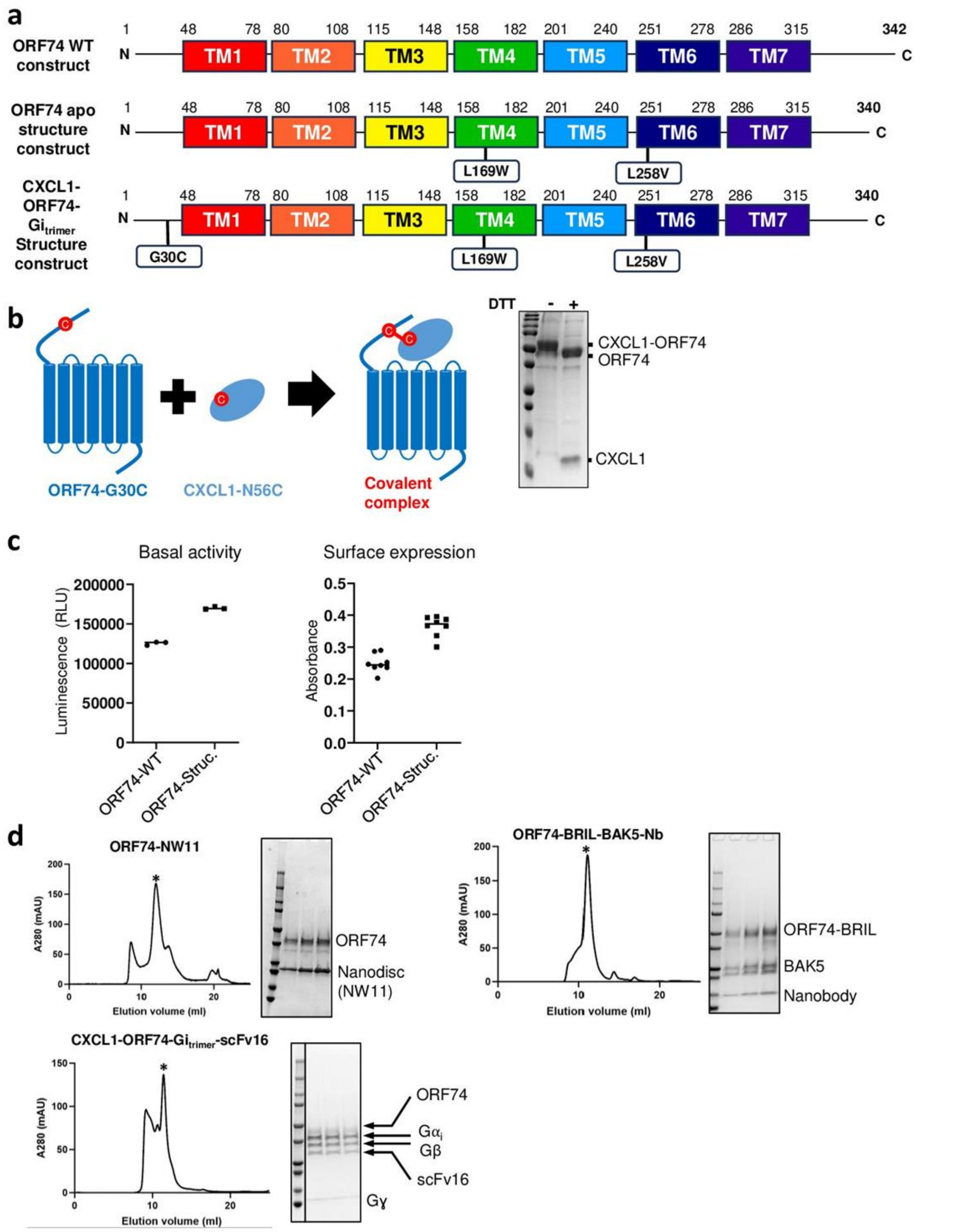

Supplementary Figure 1. **Construct design, purification and functional assay results for ORF74.** **a**, Structural study construct design. **b**, Schematic representation of the disulfide cysteine trap between ORF74 and CXCL1, with experimental validation using the redox reagent dithiothreitol (DTT). **c**, Functional comparison between wild-type ORF74 and the mutant form engineered for structural studies. The relative activity was calculated by dividing the activity values by the surface expression levels,

followed by normalization to 1 using the ORF74 WT value. The basal activity test represents four independent experiments (n = 4), and the surface expression test represents eight independent experiments (n = 8). **d**, Size exclusion chromatography elution profiles and coomassie blue-stained SDS-PAGE images of ORF74 variants. The asterisk denotes the peak fraction used for cryoEM grid preparation.

Figure S2

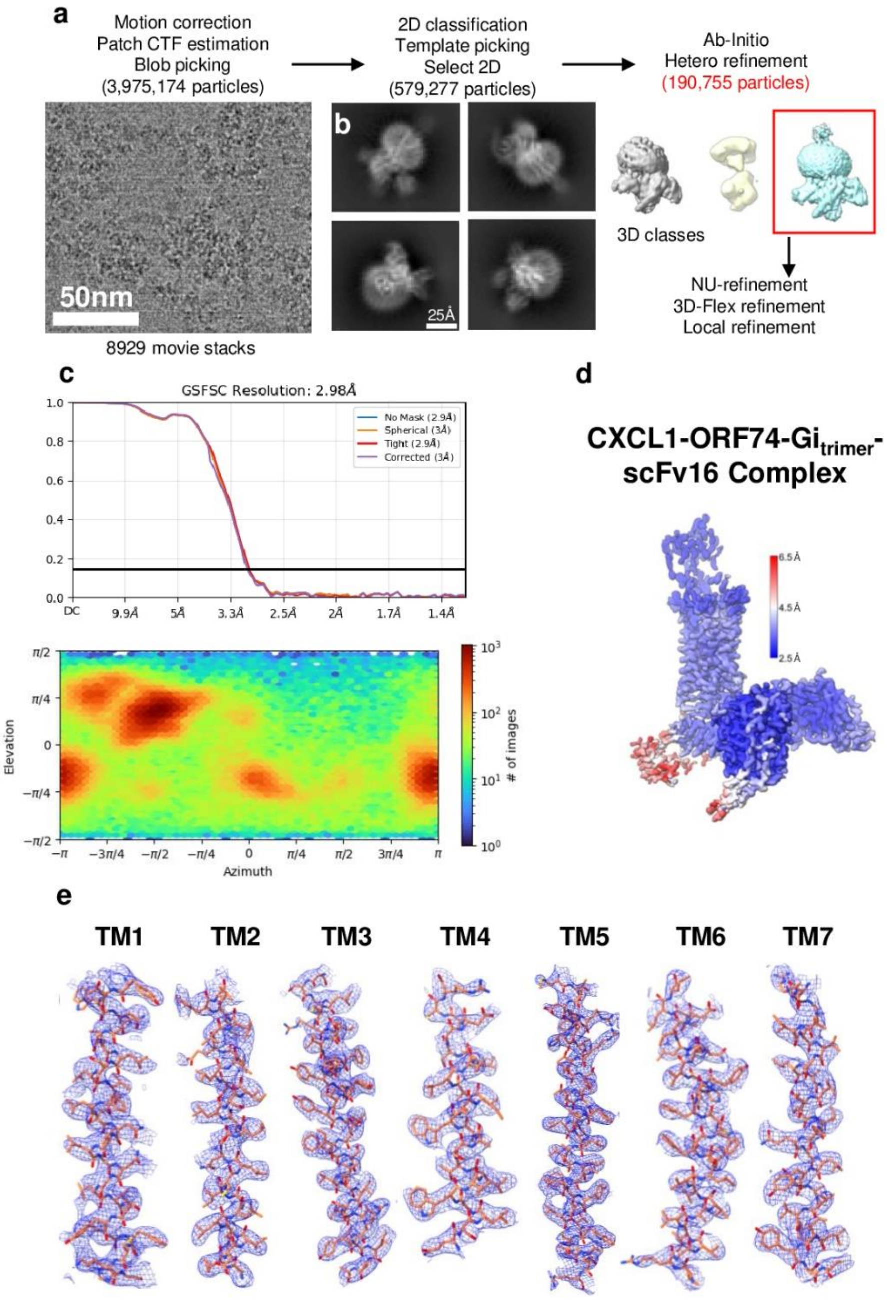

Supplementary Figure 2. CryoEM structural characterization of CXCL1-ORF74-Gi$_{trimer}$-scFv16. a, Overall cryoEM data processing procedure, representative cryo-micrograph and **b**, 2D class averages of the CXCL1-ORF74-Gi$_{trimer}$-scFv16 sample. **c**, Top panel - Gold standard Fourier shell correlation (GSFSC) plot for the 3DFlex map and bottom panel - orientation distribution plot for particles (n = 190,755) used in the consensus 3D reconstruction. **d**, Local resolution of the 3DFlex cryoEM

density map. **e**, High resolution information of the TM regions from overlay of the CXCL1-ORF74-Gi$_{trimer}$-scFv16 composite cryoEM density map (contour level = 0.4) and model.

Figure S3

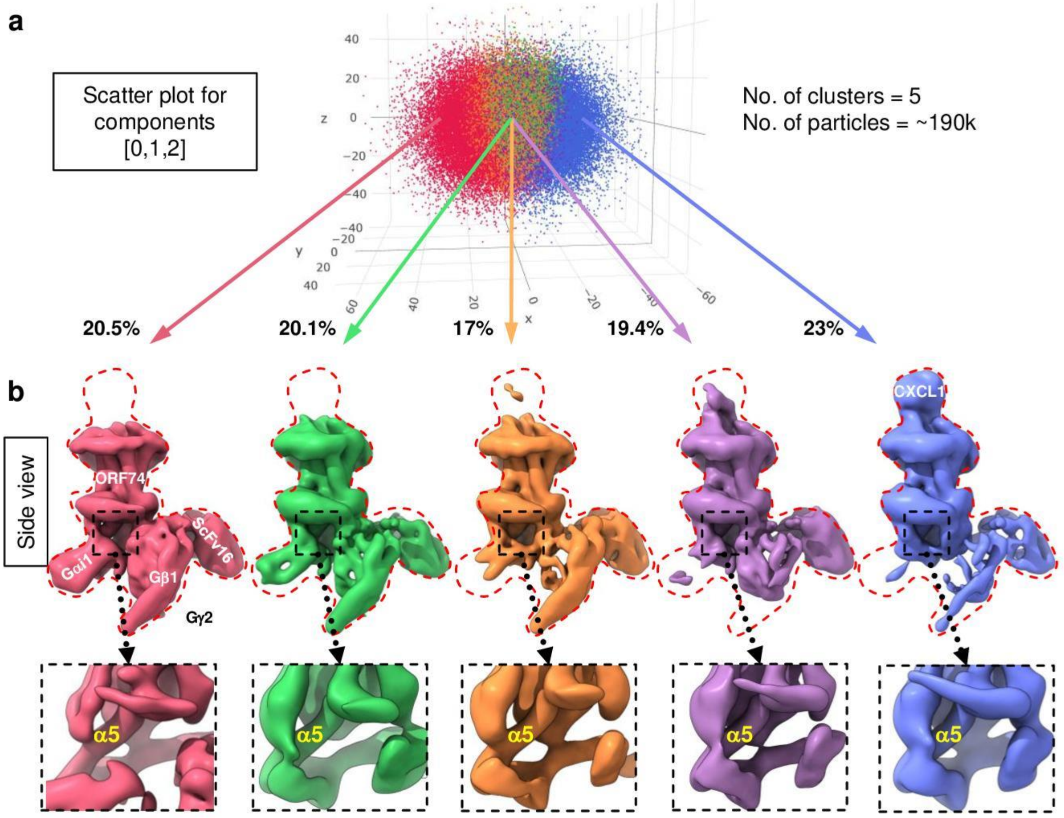

Supplementary Figure 3. 3D variability analysis of the CXCL1-ORF74-Gi$_{trimer}$-scFv16 cryoEM data. **a**, Clustered scatter plot of the 3D variability (3DV) analysis, generated using a 3DV display job. **b**, Top panel – Consensus maps color-coded for each of the 5 clusters are shown. The percentage of particles distributed in each class is also indicated. The red dashed line marks the boundary of the overall map density. Bottom panel – Zoomed-in view of the Gαi/α5 helix docking into the intracellular pocket of the ORF74/7TM regions.

Figure S4

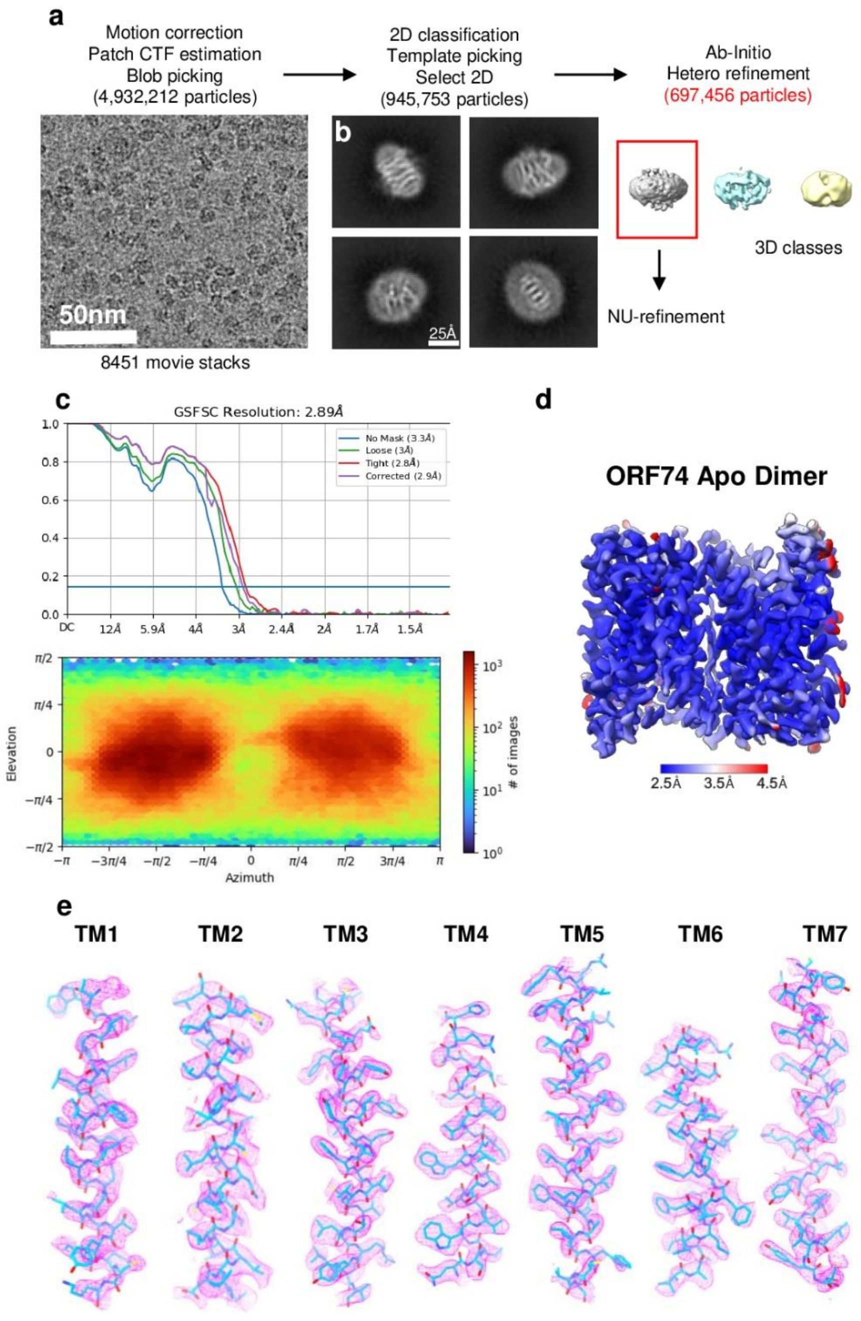

Supplementary Figure 4. CryoEM structural characterization of KSHV ORF74 in the apo conformation. a, Overall cryoEM data processing procedure, representative cryo-micrograph and **b**, 2D class averages of the ORF74 sample. **c**, Top panel - GSFSC plot and bottom panel - orientation distribution plot for the particles (n = 697,456) used in the final 3D reconstruction. **d**, Local resolution of the cryoEM density map. **e**, High resolution information of the TM regions from the ORF74 Apo cryoEM density map (contour level = 0.5).

Figure S5

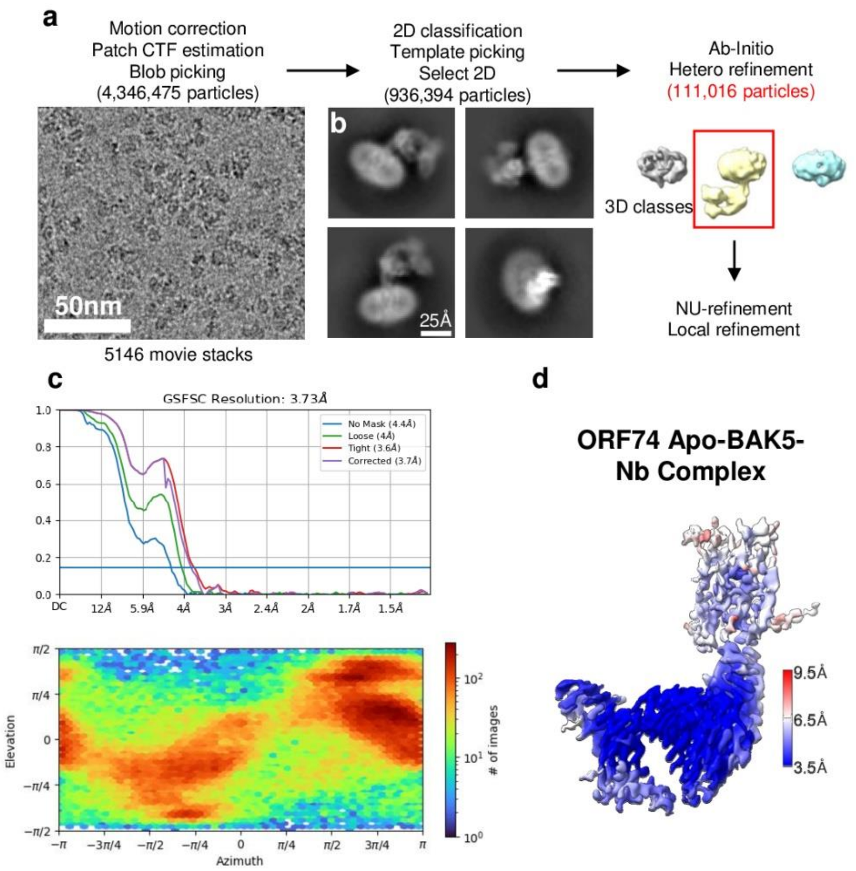

Supplementary Figure 5. **CryoEM structural characterization of ORF74-BRIL. a**, Overall cryoEM data processing procedure, representative cryo-micrograph and **b**, 2D class averages of the ORF74 sample. **c**, Top panel - Gold standard Fourier shell correlation (GSFSC) plot and bottom panel - orientation distribution plot for the particles (n = 190,755) used in the final 3D reconstruction. **d**, Local resolution of the cryoEM density map.

Figure S6

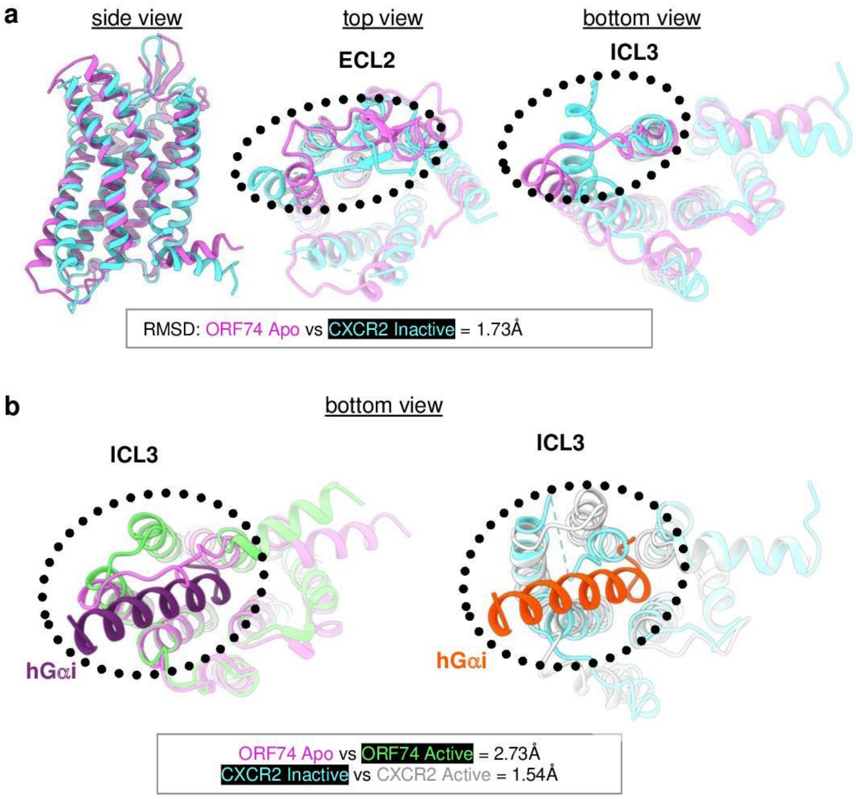

Supplementary Figure 6. **Structural comparison with emphasis on ECL2 and ICL3. a**, Comparative structural analysis between apo ORF74 and inactive CXCR2, focusing on the ECL2 and ICL3 regions. **b**, Structural comparison between ORF74 in its apo (inactive) and active conformations (left panel), which are juxtaposed with CXCR2 in its inactive and active states.

Figure S7

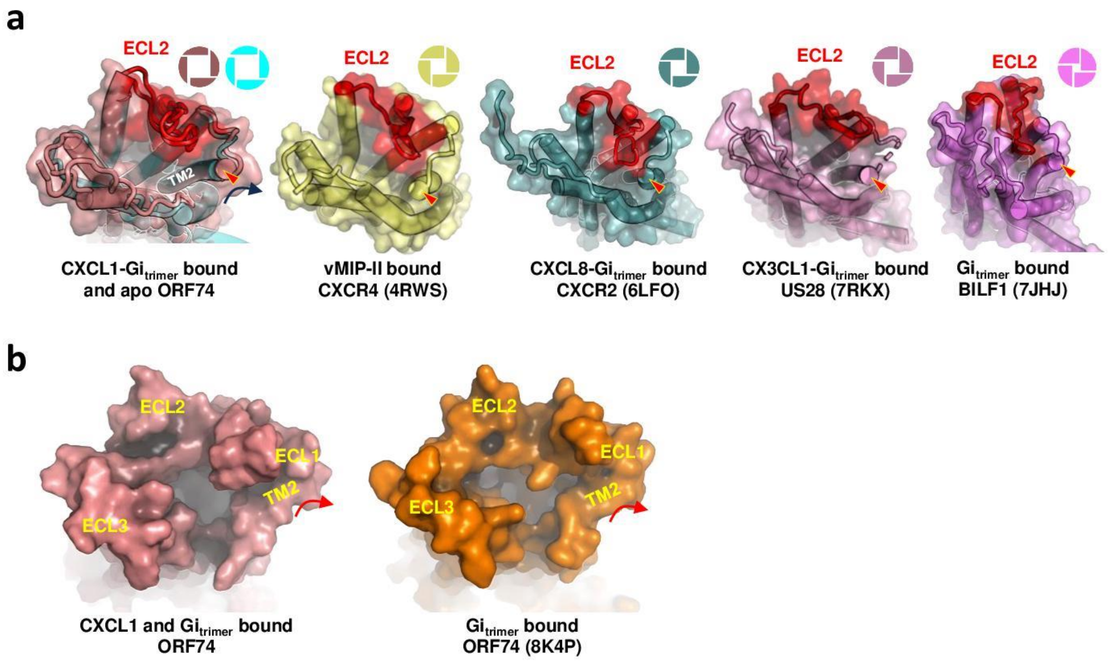

Supplementary Figure 7. Structural comparison of the ligand binding pocket. a, Structural comparison of the same orientation of the extracellular ligand binding pocket and ECL2 in the ligand-bound conformations of GPCRs. TM2 is indicated by a red arrowhead within a yellow frame, whereas ECL2 is represented in red. The PDB codes for the structures of vMIP-II bound CXCR4, CXCR8 bound CXCR2, CX3CL1 bound US28, and BILF1 are 4RWS, 6LFO, 4XT1, and 7JHJ, respectively. **b,** Comparison of the ligand binding pocket between ORF74 bound to CXCL1/Gi$_{trimer}$ and ORF74 bound to Gi$_{trimer}$ alone.

Figure S8

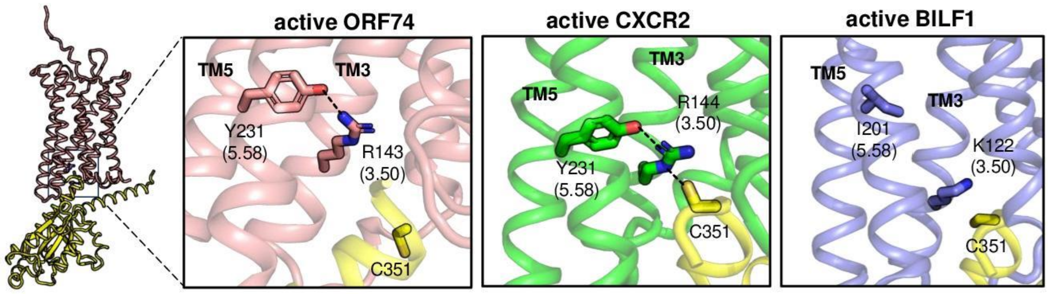

Supplementary Figure 8. **Structural comparison focused on the TM3-TM5-$G_{\alpha i}$ interaction.** Structural comparison of interactions between $G_{\alpha i}$ Cys351 (yellow cartoon) and residues at positions 5.58 and 3.50 in the active conformations of ORF74 (brown cartoon), CXCR2 (green cartoon), and BILF1 (purple cartoon).

Figure S9

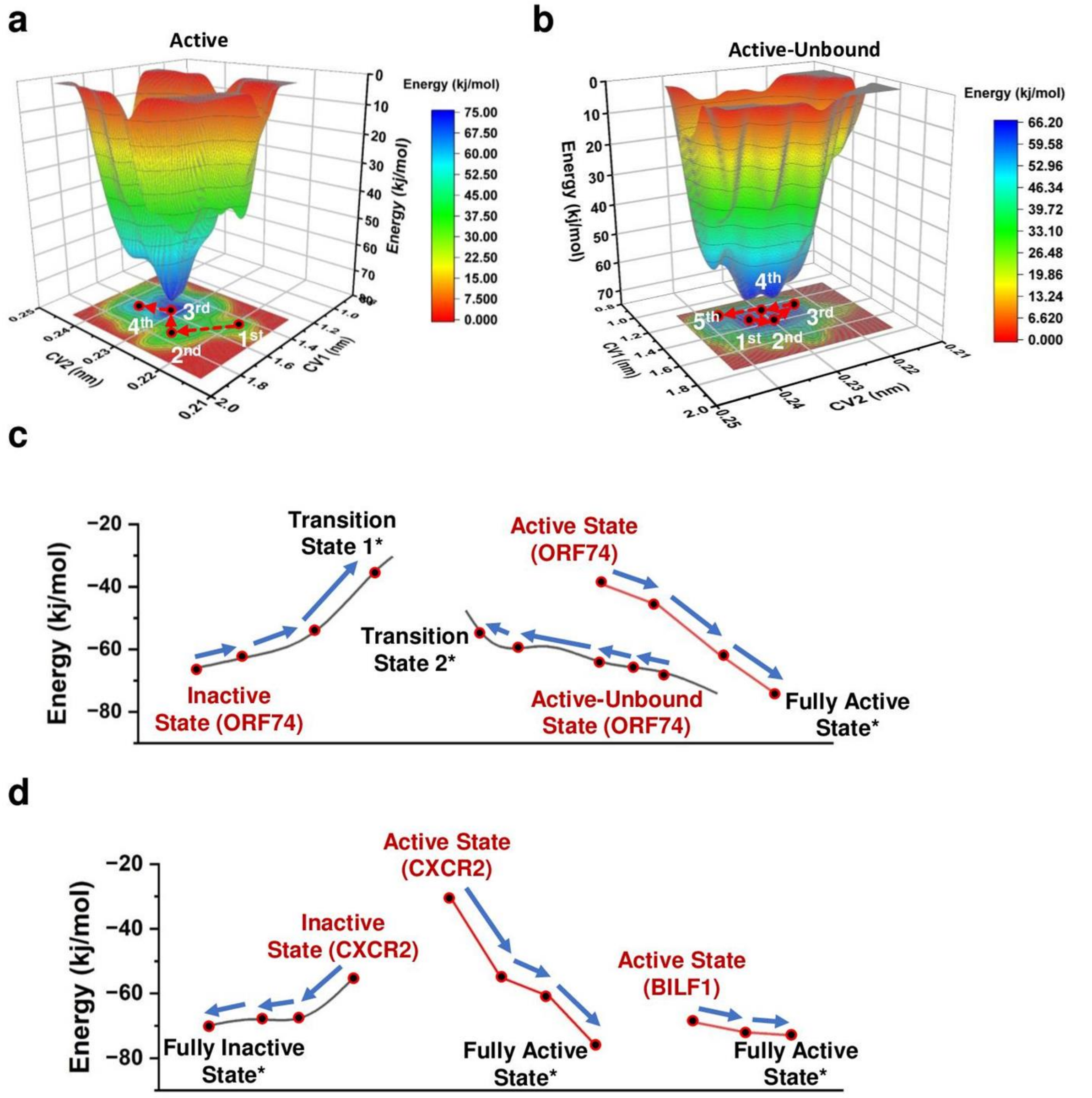

Supplementary Figure 9. Free energy landscapes from metadynamics simulations for various conformational states of ORF74, CXCR2, and BILF1. **a**, Free energy landscape of active-bound ORF74 showing a downhill movement toward lower-energy active conformations. **b**, Active-unbound ORF74 movement toward a distinct higher energy intermediate (denoted as transition state 2), in the direction of the inactive state. **c**, Comparison of overall energy landscapes for ORF74 conformers: the inactive and active-unbound states movement toward distinct high-energy intermediates, while the active-bound state stabilizes in a low-energy active conformation. **d**, Comparison with CXCR2 and BILF1: active-bound forms behave similarly to ORF74, while the inactive CXCR2 shows a downhill shift, indicating a more stable inactive state and less conformational flexibility. Only the energy minima values from the 3D free-energy landscapes of CXCR2 and BILF1 are shown in this plot. The full 3D landscapes are not included. Each point represents an energy minimum within a funnel for the respective simulation trajectory.

Figure S10

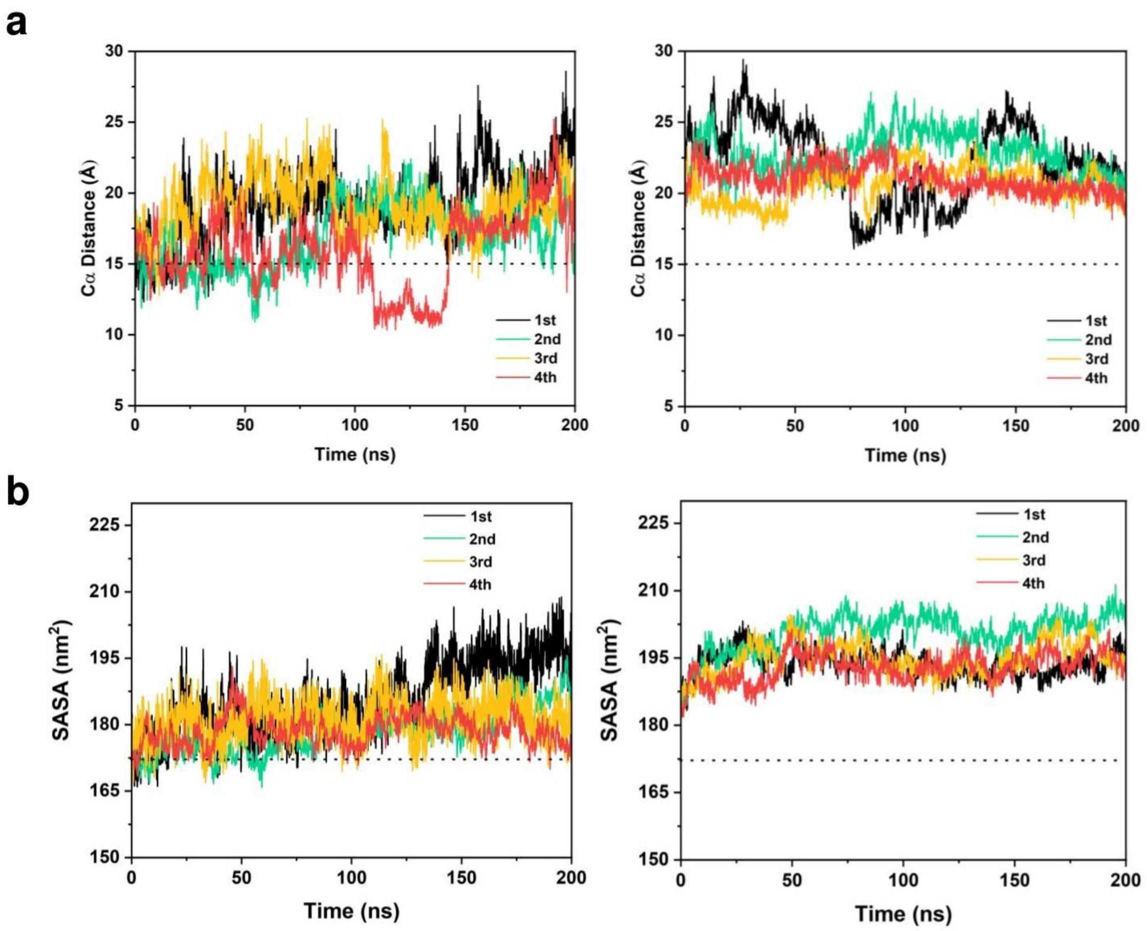

Supplementary Figure 10. Conformational Transitions in ORF74 Captured Across Metadynamics Replicas. **a**, TM3–TM6 Cα distance across metadynamics replicas of inactive (left) and active (right) ORF74. **b**, Solvent Accessible Surface Area (SASA) changes across metadynamics replicas of inactive (left) and active (right) ORF74. Across all replicas of the inactive ORF74 simulations, we observed a consistent increase in the TM3–TM6 distance and SASA.

Figure S11

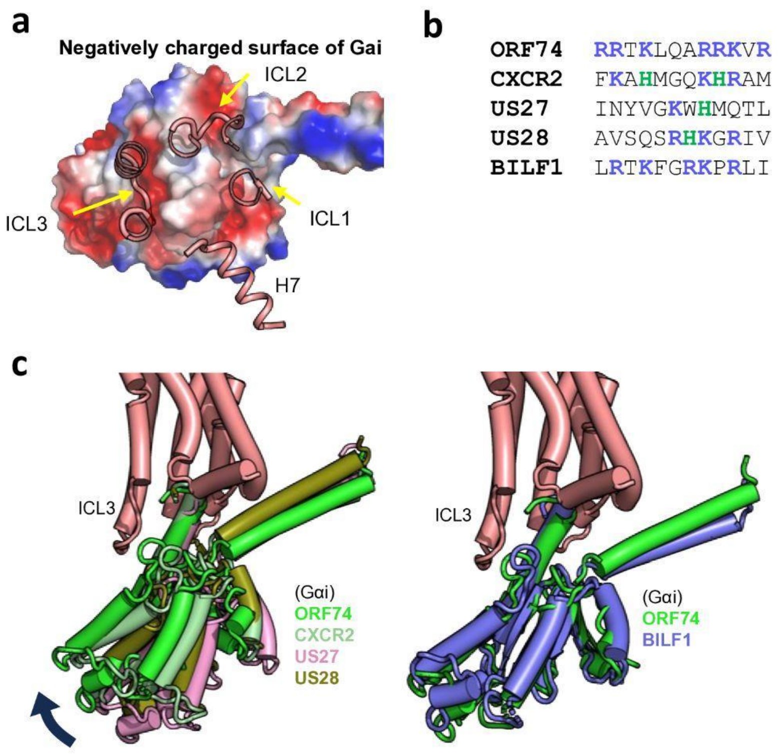

Supplementary Figure 11. **Interaction between the intracellular part of GPCRs and $G_{\alpha i}$. a**, Surface charge distribution of $G_{\alpha i}$ and its interaction interface with the intracellular domain of ORF74. **b**, Amino acid sequence alignment of the ICL3 region of ORF74, CXCR2, US27, US28, and BILF1. Positively charged arginine and lysine residues are highlighted in yellow. **c**, Characteristic tilting of the $G_{\alpha i}$ toward ICL3 of ORF74 (left panel). A similar structural feature is observed in BILF1 (right panel).

Figure S12

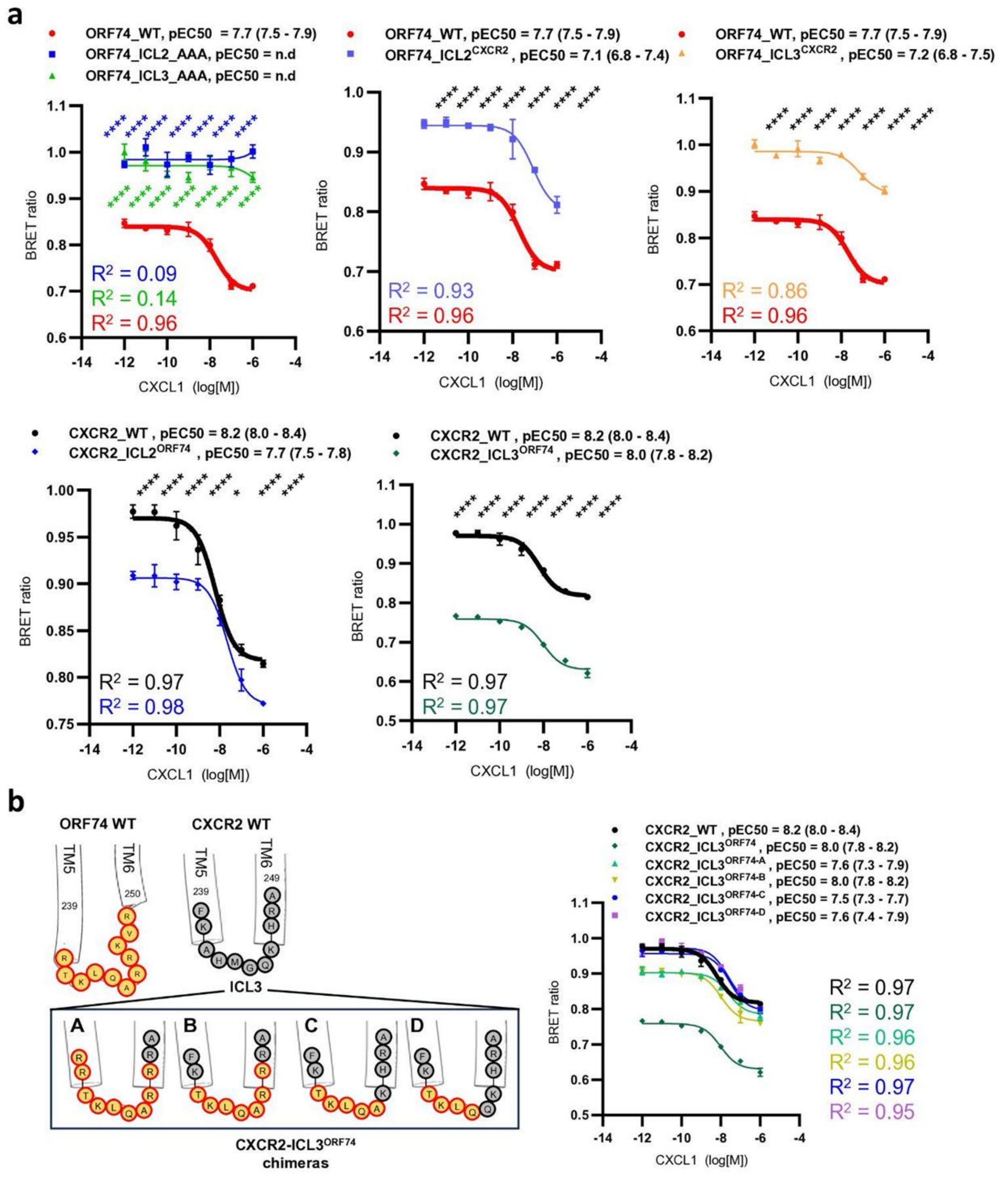

Supplementary Figure 12. ICL2 and ICL3 of ORF74 roles in its G protein signaling. **a**, BRET changes in ORF74 wild-type (WT) and ORF74 chimeras, as well as CXCR2 wild-type and CXCR2 chimeras. Wild-type trendlines highlighted in bold. ORF74_ICL2_AAA and ORF74_ICL3_AAA indicate constructs in which each respective ICL was replaced with alanines. **b**, Schematic representation of different CXCR2-ICL3^{ORF74} chimera designs (left panel) and a comparison of their BRET signals relative to wild-type (right panel). R^2 and pEC50 values were calculated using GraphPad Prism. The data represents four independent experiments ($n = 4$), with bars indicating mean ± SEM (****$p < 0.0001$, ***$p < 0.001$, **$p < 0.01$, *$p < 0.05$, ns > 0.05). Significance was determined using ordinary one-way

ANOVA. Detailed statistical methods are described in the Methods section and raw data are provided in the Source Data file.

Figure S13

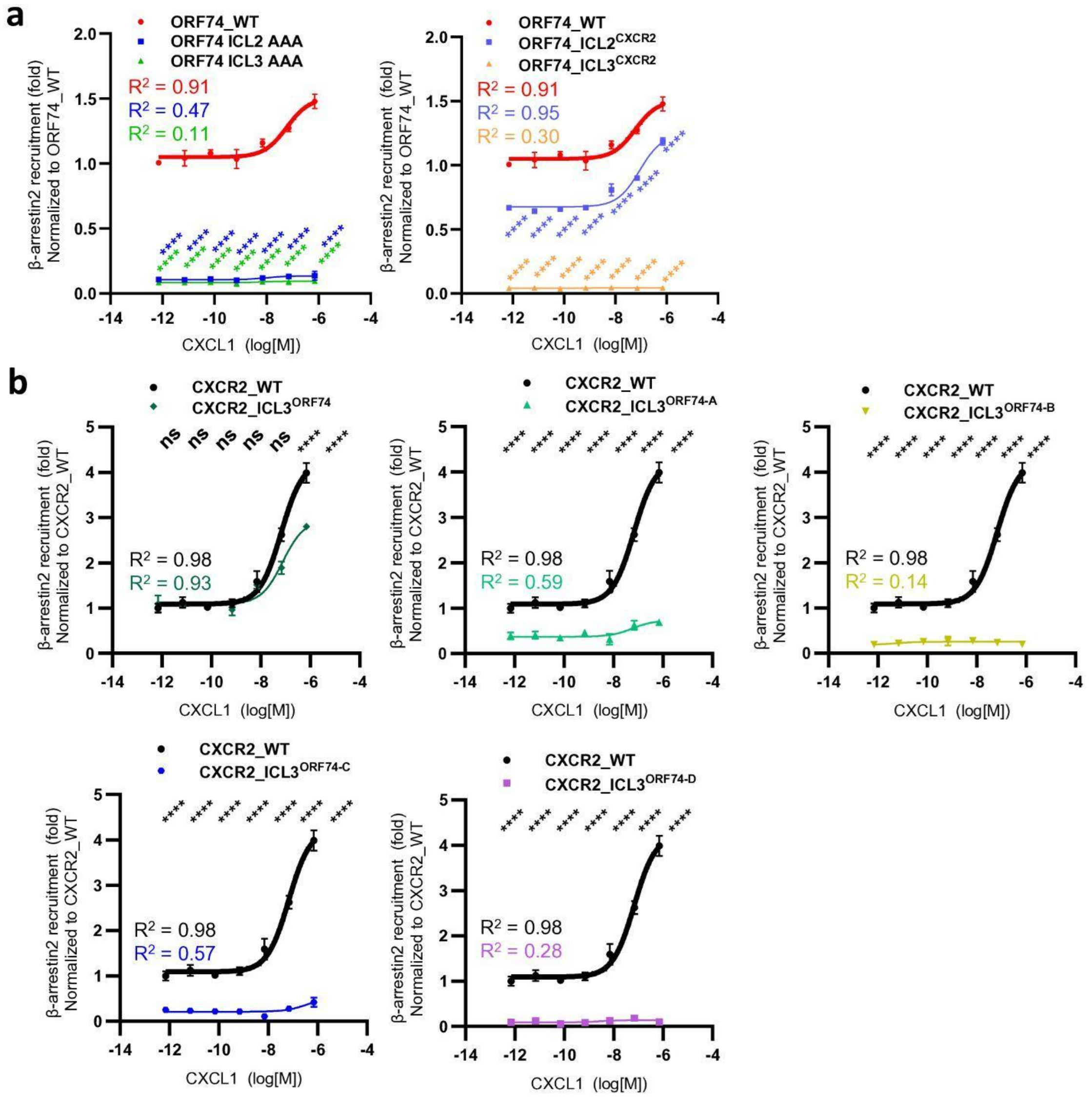

Supplementary Figure 13. Comparison of the effects of ORF74 ICL2 and ICL3 on β-arrestin recruitment. a, β-arrestin recruitment assay (PRESTO-Tango) for ORF74 wild-type (WT) and chimeras. Data represents two independent experiments (n = 2), except for ORF74_ICL2 AAA and ORF74_ICL3 AAA, which were tested in three independent experiments (n = 3). **b**, β-arrestin recruitment assay for CXCR2 wild-type and chimeras, with wild-type trendlines highlighted in bold (data represent three independent experiments, n = 3). ORF74_ICL2 AAA and ORF74_ICL3 AAA are constructs in which the respective ICLs were replaced with alanines. Relative light units (RLU), indicating β-arrestin recruitment, were normalized to CXCR2 WT. Bars show mean ± SEM (****p < 0.0001, ***p < 0.001, *p < 0.01, p < 0.05, ns > 0.05). Significance was determined by ordinary one-way ANOVA. Detailed statistical methods are described in the Methods section, and raw data are provided in the Source Data file.

Figure S14

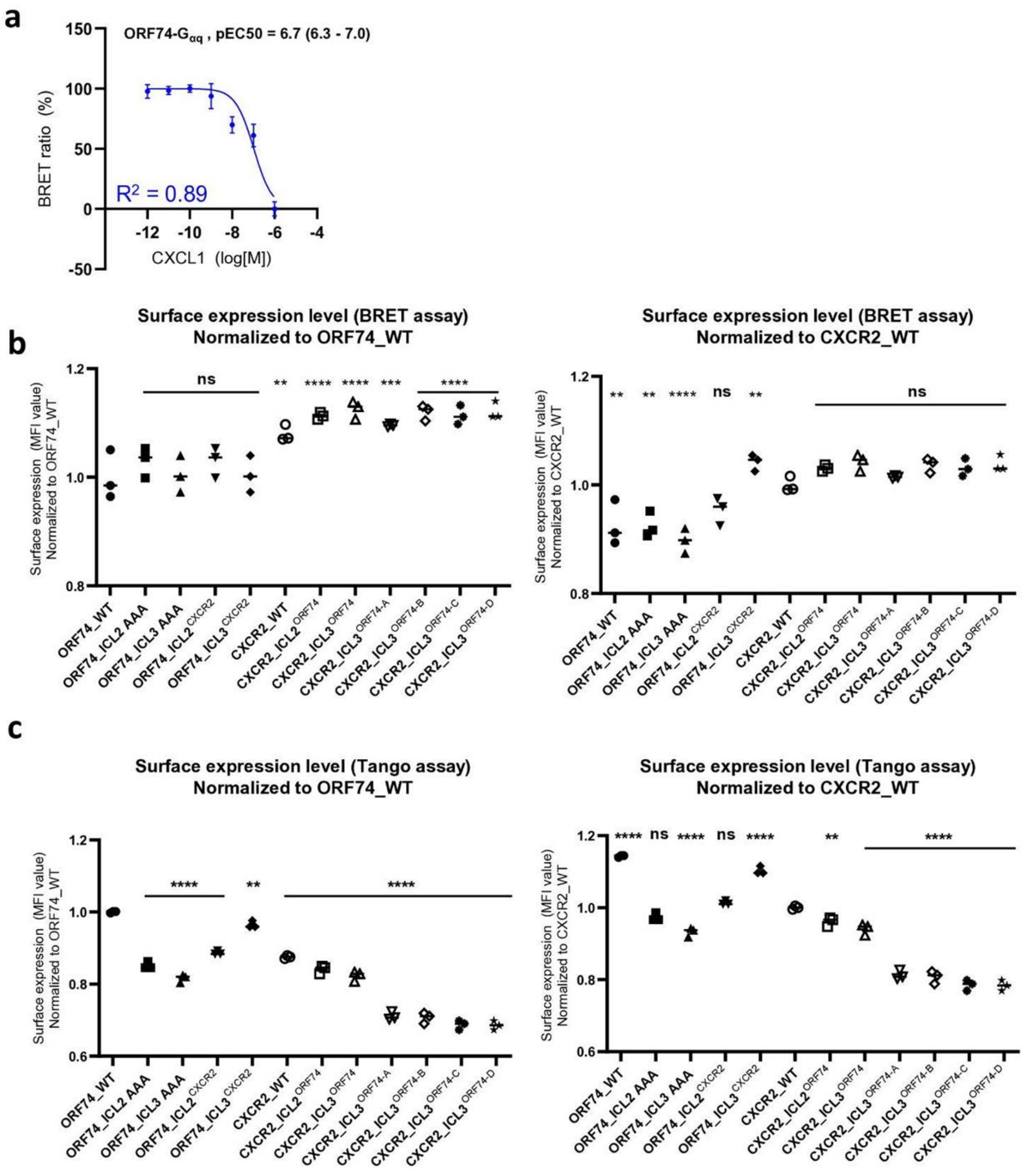

Supplementary Figure 14. Comparison of EC50 values for $G_{\alpha i}$ and $G_{\alpha q}$ in ORF74 and surface expression level. a, pEC50 values were determined through CXCL1 dose-dependent BRET signal analysis of ORF74 with $G_{\alpha q}$. The data represents four independent experiments (n = 4). pEC50 values were calculated using GraphPad Prism. pEC50 for $G_{\alpha q}$ could not be determined due to the absence of saturation activity at high CXCL1 concentrations. **b**, Comparison of surface expression levels among ORF74, CXCR2, and their respective chimeras for the BRET assay in HEK293 cells. The left panel shows normalization to ORF74 wild-type, while the right panel shows normalization to CXCR2 wild-type. The data represents three independent experiments (n = 3). **c**, Comparison of surface expression levels

among ORF74, CXCR2, and their respective chimeras for the Tango assay in HTLA cells (a HEK293-derived cell line stably expressing tTA-dependent luciferase reporter gene and a β-arrestin-2-TEV fusion protein). The left panel represents normalization to ORF74 wild-type, and the right panel represents normalization to CXCR2 wild-type. The data represents three independent experiments (n = 3). Bars indicating mean ± SEM (****$p < 0.0001$, ***$p < 0.001$, **$p < 0.01$, *$p < 0.05$, ns > 0.05). Significance was determined using ordinary one-way ANOVA. Detailed statistical methods are described in the Methods section and raw data are provided in the Source Data file.

Supplementary Table 1. CryoEM Data Collection, Refinement and Validation statistics

	ORF74 Apo Dimer (EMD-43717) (PDB 8W1A)	ORF74-BRIL-BAK5-Nb Apo Monomer (EMD-43720)	CXCL1-ORF74-Gi$_{trimer}$-scFv16 Complex			
			Composite map (3DFlex+local refined map) (EMD-48100) (PDB 9EJC)	NU-refined consensus map (EMD-43718)	3DFlex refined map (EMD-48095)	CXCL1-ORF74 local refined map (EMD-48097)
**Data collection and processing**						
Magnification	130,000×	130,000×	130,000×			
Voltage (kV)	300	300	300			
Electron exposure (e/Å^2)	50	50	50			
Defocus range (μm)	-0.8 to -1.5	-0.8 to -1.5	-0.8 to -1.5			
Pixel size (Å)	0.66	0.66	0.66			
Symmetry imposed	C1	C1	C1			
Initial particle images (no.)	4,932,212	4,346,475	3,975,174			
Final particle images (no.)	697,456	111,016	190,755			
Map resolution (Å)	2.89	3.73	N/A	3.06	2.98	3.61
FSC threshold	0.143	0.143		0.143	0.143	0.143
Auto Sharpening/ Post processing	B factor (Å^2) -130.4	B factor (Å^2) -142.0		DeepEMhancer		
**Refinement**						
Initial model used (PDB code)	AlphaFold2 predicted ORF74	N/A	AlphaFold2 predicted ORF74 and 7JHJ	N/A		
Model resolution (Å)<br>FSC threshold	3.2<br>0.5		3.3<br>0.5			
**Model composition**<br>Non-hydrogen atoms<br>Protein residues<br>Ligands	 4,664<br>584<br>0		 9,485<br>1,214<br>0			
**R.M.S. deviations**<br>Bond lengths (Å)<br>Bond angles (°)	 0.003<br>0.573		 0.002<br>0.481			
**Validation**<br>MolProbity score<br>Clash score<br>Rotamer outliers (%)	 1.28<br>4.08<br>0		 1.43<br>3.16<br>0			
**Ramachandran plot**<br>Favored (%)<br>Allowed (%)<br>Disallowed (%)	 97.59<br>2.41<br>0.00		 95.57<br>4.43<br>0.00			
