# Supplementary material for: Structural basis for ligand promiscuity and high signaling activity of Kaposi’s Sarcoma-associated Herpesvirus-encoded GPCR
Source: Nat Commun. 2025 Sep 25;16:8403. doi: 10.1038/s41467-025-63457-4 (PMC12462484; doi:10.1038/s41467-025-63457-4)
Supplement: Supplementary file 2 — Reporting Summary [file 41467_2025_63457_MOESM2_ESM.pdf]

nature portfolio

Corresponding author(s): Matthias Buck, Xinghong Dai and Jae U. Jung

Last updated by author(s): Jul 31, 2025

Reporting Summary

Nature Portfolio wishes to improve the reproducibility of the work that we publish. This form provides structure for consistency and transparency in reporting. For further information on Nature Portfolio policies, see our Editorial Policies and the Editorial Policy Checklist.

Statistics

For all statistical analyses, confirm that the following items are present in the figure legend, table legend, main text, or Methods section.

n/a	Confirmed	
☐	☒	The exact sample size (*n*) for each experimental group/condition, given as a discrete number and unit of measurement
☐	☒	A statement on whether measurements were taken from distinct samples or whether the same sample was measured repeatedly
☒	☐	The statistical test(s) used AND whether they are one- or two-sided<br>*Only common tests should be described solely by name; describe more complex techniques in the Methods section.*
☒	☐	A description of all covariates tested
☐	☒	A description of any assumptions or corrections, such as tests of normality and adjustment for multiple comparisons
☐	☒	A full description of the statistical parameters including central tendency (e.g. means) or other basic estimates (e.g. regression coefficient) AND variation (e.g. standard deviation) or associated estimates of uncertainty (e.g. confidence intervals)
☐	☒	For null hypothesis testing, the test statistic (e.g. *F*, *t*, *r*) with confidence intervals, effect sizes, degrees of freedom and *P* value noted<br>*Give P values as exact values whenever suitable.*
☒	☐	For Bayesian analysis, information on the choice of priors and Markov chain Monte Carlo settings
☒	☐	For hierarchical and complex designs, identification of the appropriate level for tests and full reporting of outcomes
☒	☐	Estimates of effect sizes (e.g. Cohen's *d*, Pearson's *r*), indicating how they were calculated

Our web collection on statistics for biologists contains articles on many of the points above.

Software and code

Policy information about availability of computer code

Data collection	serialEM (4.2)
Data analysis	Excel (2406), GraphPad Prism (10.1.2), CryoSPARC (4.3.1), Coot (0.9.8.92), PyMOL (2.5.7), ChimeraX (1.7), GROMACS(2021.5), PLUMED (2.8.0), MetadynView(1.0).

For manuscripts utilizing custom algorithms or software that are central to the research but not yet described in published literature, software must be made available to editors and reviewers. We strongly encourage code deposition in a community repository (e.g. GitHub). See the Nature Portfolio guidelines for submitting code & software for further information.

Data

Policy information about availability of data

All manuscripts must include a data availability statement. This statement should provide the following information, where applicable:

- Accession codes, unique identifiers, or web links for publicly available datasets
- A description of any restrictions on data availability
- For clinical datasets or third party data, please ensure that the statement adheres to our policy

The cryoEM maps have been deposited in the Electron Microscopy Data Bank (EMDB) under accession codes EMD-43717 [https://www.ebi.ac.uk/pdbe/entry/emdb/EMD-43717] (KSHV ORF74 apo dimer); EMD-48100 [https://www.ebi.ac.uk/pdbe/entry/emdb/EMD-48100] (CXCL1-KSHV ORF74-Gitrimer-scFv16 complex); EMD-43718 [https://www.ebi.ac.uk/pdbe/entry/emdb/EMD-43718] (NU-refined consensus map of CXCL1-KSHV ORF74-Gitrimer-scFv16 complex); EMD-48095 [https://www.ebi.ac.uk/pdbe/entry/emdb/EMD-48095] (3DFlex refined map of CXCL1-KSHV ORF74-Gitrimer-scFv16 Complex); EMD-48097 [https://www.ebi.ac.uk/

pdbe/entry/emdb/EMD-48097] (Local refined cryoEM map of CXCL1-KSHV ORF74 region). The atomic coordinates have been deposited in the Protein Data Bank (PDB) under accession codes 8W1A [https://doi.org/10.2210/pdb8W1A/pdb] (KSHV ORF74 apo dimer); 9EJC [https://doi.org/10.2210/pdb9EJC/pdb] (CXCL1-KSHV ORF74-Gitrimer-scFv16 complex).

Research involving human participants, their data, or biological material

Policy information about studies with human participants or human data. See also policy information about sex, gender (identity/presentation), and sexual orientation and race, ethnicity and racism.

Reporting on sex and gender	N/A
Reporting on race, ethnicity, or other socially relevant groupings	N/A
Population characteristics	N/A
Recruitment	N/A
Ethics oversight	N/A

Note that full information on the approval of the study protocol must also be provided in the manuscript.

Field-specific reporting

Please select the one below that is the best fit for your research. If you are not sure, read the appropriate sections before making your selection.

[x] Life sciences [ ] Behavioural & social sciences [ ] Ecological, evolutionary & environmental sciences

For a reference copy of the document with all sections, see nature.com/documents/nr-reporting-summary-flat.pdf

Life sciences study design

All studies must disclose on these points even when the disclosure is negative.

Sample size	The structural data for biological macromolecules are validated by physical and chemical laws, instead of statistics. The biological sample size were indicated in the text.
Data exclusions	For structural experiments shown in the manuscript, all of the data were used. All the biochemical data were presented in the figure.
Replication	All the experiments have been repeated for at least three times, with a representative result shown in the figure. All the replications attemps are successful.
Randomization	For single particle analysis of EM, samples were allocated into experimental groups randomly.
Blinding	Blinding is not necessary or valid for the purposes of structure determination.

Reporting for specific materials, systems and methods

We require information from authors about some types of materials, experimental systems and methods used in many studies. Here, indicate whether each material, system or method listed is relevant to your study. If you are not sure if a list item applies to your research, read the appropriate section before selecting a response.

Materials & experimental systems

n/a	Involved in the study	
[ ]	[x]	Antibodies
[ ]	[x]	Eukaryotic cell lines
[x]	[ ]	Palaeontology and archaeology
[x]	[ ]	Animals and other organisms
[x]	[ ]	Clinical data
[x]	[ ]	Dual use research of concern
[x]	[ ]	Plants

Methods

n/a	Involved in the study	
[x]	[ ]	ChIP-seq
[x]	[ ]	Flow cytometry
[x]	[ ]	MRI-based neuroimaging

Antibodies

Antibodies used	scFv16 (GPCR/G-protein complex stabilizer)

Validation	We used exactly same amino acid sequence of scFv16 from the paper (https://doi.org/10.1038/s41467-018-06002-w) and commercial product (https://www.thermofisher.com/antibody/product/GPCR-G-protein-complex-stabilizing-scFv-Antibody-clone-scFv16-Recombinant-Monoclonal/703976).

Eukaryotic cell lines

Policy information about cell lines and Sex and Gender in Research

Cell line source(s)	HEK293(ATCC, CRL-1573), HEK293S(GnTi-) (ATCC, CRL-3022), ExpiSf9 (ThermoFisher, A35243), Hi5 (ThermoFisher, B85502).
Authentication	All cell lines were purchased and used from each manufacturer.
Mycoplasma contamination	No mycoplasma was observed.
Commonly misidentified lines (See ICLAC register)	There is no commonly misidentified lines.

Plants

Seed stocks	N/A
Novel plant genotypes	N/A
Authentication	N/A
